# Supplementary material for: Long-term droughts may drive drier tropical forests towards increased functional, taxonomic and phylogenetic homogeneity
Source: Nat Commun. 2020 Jul 3;11:3346. doi: 10.1038/s41467-020-16973-4 (PMC7335099; doi:10.1038/s41467-020-16973-4)
Supplement: Supplementary file 1 — Supplementary Information [file 41467_2020_16973_MOESM1_ESM.pdf]

Long-term droughts may drive drier tropical forests towards increased functional, taxonomic and phylogenetic homogeneity

Aguirre-Gutiérrez et al.

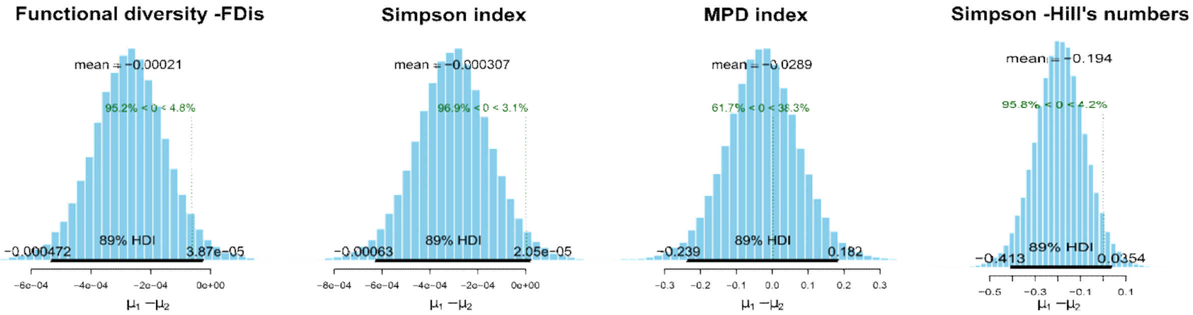

Supplementary Figure 1. Difference in rates of change in each diversity facets between dry and wet tropical forests. Posterior distribution of changes observed in functional (FDis), taxonomic (Simpson index and as Hill's numbers) and phylogenetic diversity (MPD), after standardising for time between the first and second census, between drier plots (MCWD ≤ -250 mm during second time period), and wetter plots (MCWDFull > -250 mm during second time period). Drier plots, which also experienced stronger decreases in water availability across time (larger $\Delta MCWD_{Abs}$) show on average larger decreases in functional (FDis, probability= 95.2%) and taxonomic (Simpson, probability= 96.9%) diversity than wetter forests (see inset legends for full statistical results). There is no important difference in changes between drier and wetter plots for phylogenetic diversity (MPD, probability= 61.7%). The Simpson index calculated as Hill's numbers and described in the methods section (fourth histogram) showed the same pattern of results (probability= 95.8%) than the common Simpson index (second histogram),also described in the methods section.

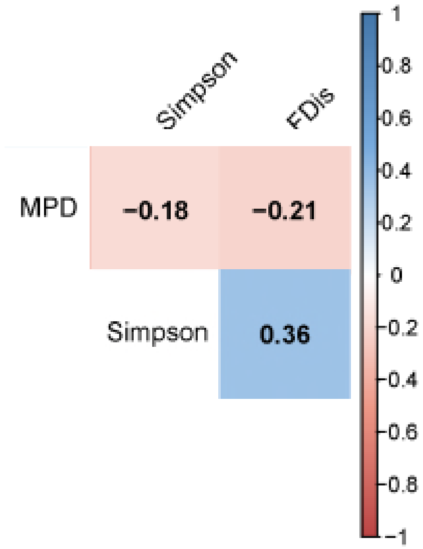

Supplementary Figure 2. Pearson's correlation analysis between the change in the three diversity facets. No significant correlations were found.

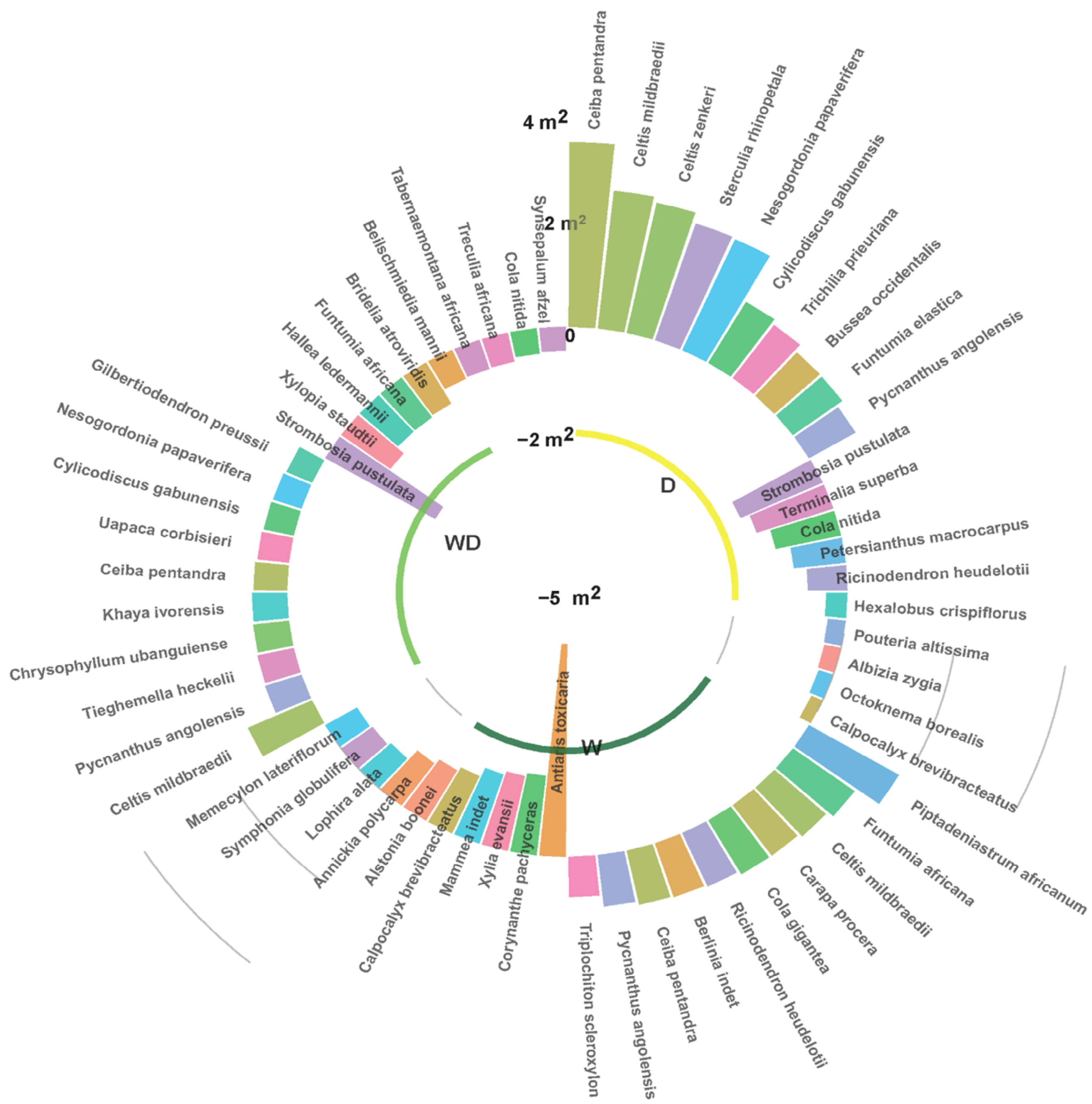

Supplementary Figure 3. The 10 species with strongest increases and decreases in Basal Area (BA m^2) across the climatic gradient. Here the forest plots were grouped as dry (D) which had an MCWDFull<-256 mm, humid (WD) with MCWDFull below -211 and above-256 mm or wet (W) with MCWDFull >-211 mm. Each vertical bar represents a species and the colours represent the same species across forest communities. The drier forests communities (D) have some of the species that have experienced the strongest increases in BA across the climatic gradient. The wettest communities (W) seem to have some of the species with the strongest decreases in BA, meanwhile BA changes in the humid forests (WD) have been milder than for the W and D communities.

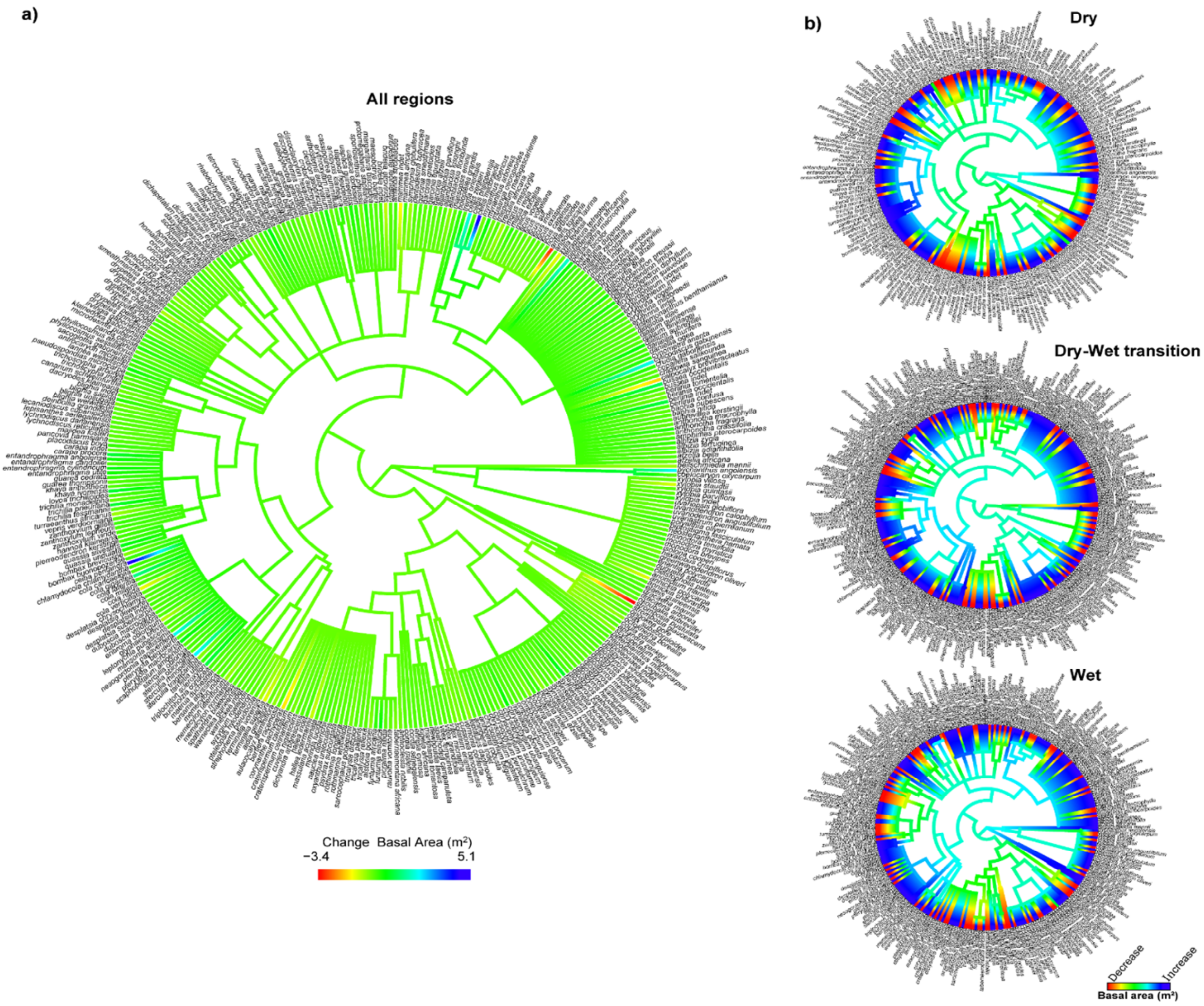

Supplementary Figure 4. Changes in basal area (m^2) along the phylogenetic tree. a) Changes in basal area (m2) per species across the full set of vegetation plots. Stronger basal area changes are depicted in light-dark blue (increases in basal area) and yellow-red (decreases in basal area), with species in green showing small basal area changes. b) Changes in basal area per species and per forest region based on the climatic water deficit of the vegetation plots. Here the forest plots were grouped as 'Dry' which had a full term maximum climatic water deficit (MCWDFull) < -256mm, 'Dry-Wet' transition with MCWDFull < -211 and > -256mm or wet (W) with MCWDFull > -211mm. For each species (the tips of the tree), basal area increases (independently of their magnitude) are shown in dark blue and decreases in red. Light blue to yellow colour ranges depict the average change in basal area for each specific clade.

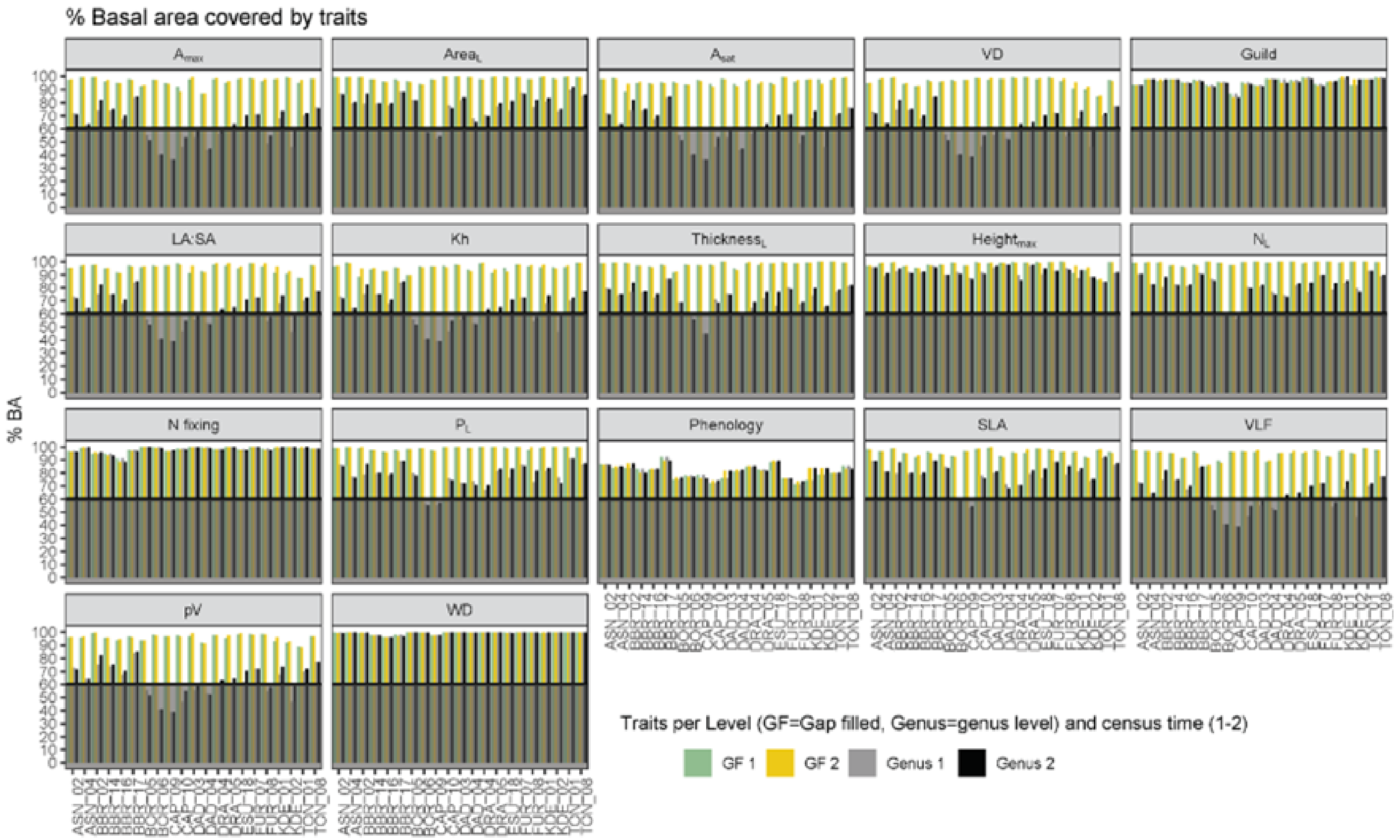

Supplementary Figure 5. Percentage basal area covered by trait information per vegetation plot. Genus 1= genus level trait representation during the first census; Genus 2= genus level trait representation for the second vegetation census. GF 1= trait gap filled dataset for vegetation census 1 and GF 2 is for vegetation census 2 (from Aguirre-Gutiérrez et al. 2019). For comparison purposes on the amount of trait data available between plots the horizontal black line is shows the 60% BA representation per plot.

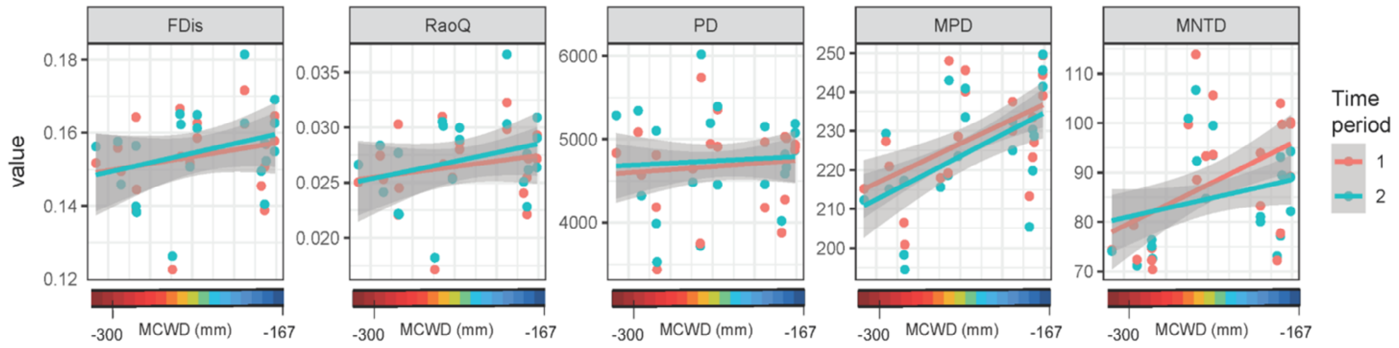

Supplementary Figure 6. Comparison of different functional and phylogenetic diversity metrics. The functional diversity metrics are FDis: functional dispersion and RaoQ. The phylogenetic diversity metrics are PD: phylogenetic distance, MPD: mean phylogenetic distance and MNTD: mean nearest taxon distance. Coloured lines are the average fit of a linear model relating each of the diversity metrics and the maximum climatic water deficit (MCWD) per plot (*n* = 21) and time period. Grey shading shows their 95% confidence intervals.

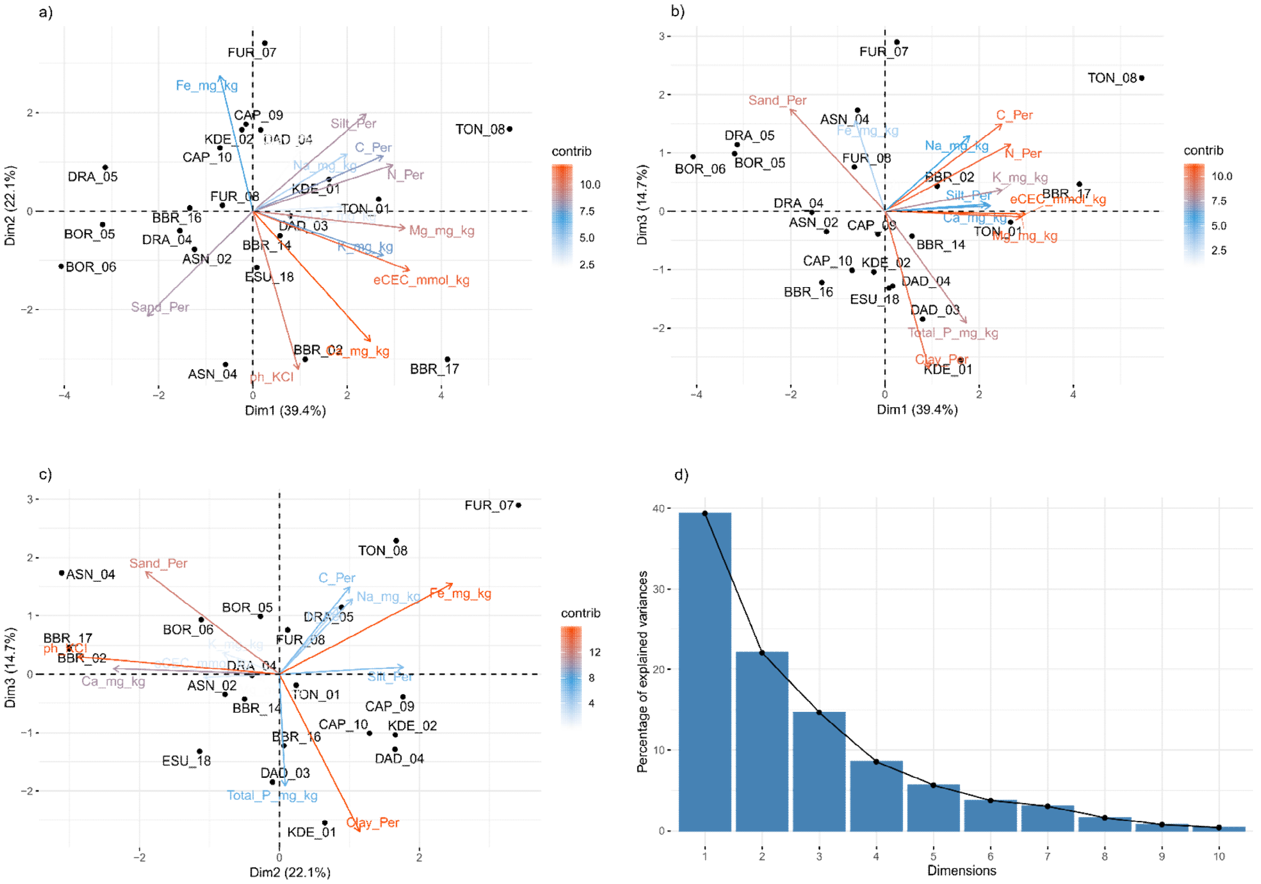

Supplementary Figure 7. Results of the Principal Component Analysis. The first three axis were selected, which together explain 76.2% of the variance in the soil data. a) shows the first and second axes, b) the first and third axes and c) the second and third axes. d) shows the percentage of variance explained by each axis in the PCA.

Supplementary discussion on forest community dynamics

To characterise the dynamics at the community level we calculated the changes in the species' basal area, temporal diversity metrics, such as the community turnover, species appearances and disappearances and mean rank shifts, and the variance ratio community stability metric [1, 2]. All community dynamics analyses were carried out using the "codyn" package in R platform (v3.4.1; http://cran.r-project.org)[3] .

There were large changes in species basal area along the climatic gradient and across time (Supplementary Figure 1). Forest communities at the locations with most negative maximum climatic water deficits (MCWD) showed some of the most extreme increase in basal area of species well adapted to drier conditions, such as *Nesogordonia papaverifera* and *Ceiba pentandra.* These two species increased their basal area by 2.2 and 3.4 m^2 respectively in such drier locations. The drier communities also witnessed strong decreases (up to 1.6 m^2) of species with preferences for wetter climates such as *Strombosia pustulata.* Maximum basal area increases at locations with less restricted water availability, i.e. less negative MCWD, were smaller, of up to 1.8 m^2, and maximum decreases where up to 1.5 m^2 with only one species showing a decrease of 4 m^2 (Supplementary Figure 3). When calculating basal area changes per species across all sampled plots (n=21) the species with the largest increases were *Ceiba pentandra* (5.11 m^2), *Celtis midbraedii* (5.09 m^2) and *Sterculia rhinopetala* (2.75 m^2), whereas *Strombosia pustulata* and *Antiaris toxicaria* showed the strongest total basal area decreases (-3.39 and -3.09 m^2 respectively). The species with larger increases and decreases in basal area did not appear clustered in specific locations of the phylogenetic tree that contained all species present in the studied plots (Supplementary Figure 4a). However, at the plot level (phylogenetic tree per plot), the groups/clades changing the most in basal area became more apparent but a strong variation between census plots was still observed (Supplementary Figure 4b).

The overall community dynamics with species turnover and species appearances and disappearances, mean rank shifts and variance ratio did not differ significantly (*P-val* >0.05) along the climatic gradient. Moreover, most sampling plots (12 of them) have increased their basal area with an annual rate of between 0.005 (BBR-14) and 0.36 m^2 (KDE-02) and only eight showed annual rate decreases of between -0.03 (DRA-04) to -0.21 m^2 (FUR-07). Such changes in basal area however where not significantly related to changes in functional (R^2=-0.01, *P-val*=0.94), taxonomic (R^2=0.28, *P*= 0.21) or phylogenetic diversity (R^2=-0.17, *P*= 0.46).

Supplementary Table 1. Details of the vegetation plots for which censuses and soil data was obtained. All vegetation census data was acquired from the ForestPlots.net database.

Plot code	Longitude	Latitude	Area used (ha)	Censuses years	Time lapse (years)	Soil sampling year
CAP_09	-2.0400	4.8500	1	1993-2013	20	2007
CAP_10	-2.0492	4.7978	1	1993-2013	20	2007
DRA_04	-2.3824	5.1642	1	1990-2009	19	2009
DRA_05	-2.4358	5.2109	1	1990-2009	19	2009
FUR_07	-2.3851	5.5612	1	1990-2009	19	2009
FUR_08	-2.3917	5.5799	0.6	1990-2009	19	2009
ASN_02	-2.2150	6.5570	0.6	1993-2007	14	2007
ASN_04	-2.1700	6.4750	0.88	1993-2007	14	2007
BBR_02	-1.3441	6.6796	1	1990-2010	20	2010
BBR_14	-1.2933	6.7087	0.88	1990-2010	20	2010
BBR_16	-1.2886	6.6982	0.92	1990-2010	20	2010
BBR_17	-1.2829	6.6873	0.96	1990-2010	20	2010
ESU_18	-0.8031	5.8626	0.52	1993-2010	17	2010
KDE_01	-0.9227	6.1510	0.69	1987-2010	23	2010
KDE_02	-0.9218	6.1527	0.96	1987-2010	23	2010
BOR_05	-1.8349	5.3451	1	1993-2009	16	2009
BOR_06	-1.8368	5.3538	1	1993-2009	16	2009
DAD_03	-3.0082	5.9915	1	1993-2013	20	2013
DAD_04	-3.0155	5.9916	1	1993-2013	20	2013
TON_01	-2.1186	6.0706	1	1991-2009	18	2009
TON_08	-2.1030	6.0390	1	1991-2009	18	2009

Supplementary Table 2. The hypothesised plant trait response and their importance under a climate drying context. The plant traits shown are used in the calculation of the forests functional diversity metric (FDis). References are not exhaustive.

Trait	Importance	Hypothesised response to a drying climate	References
LA:SA	Crucial for water transport, related effects on photosynthetic rate and mechanical strength. Lower values confer greater transport capacity on a leaf area basis.	The ratio may decrease as to prevent water loss. Stronger changes in more water-limited forests and thus decreases in this trait with increases in the abundance of deciduous species.	(4, 5)
K*p*	Water transport capacity; Index of hydraulic efficiency and possible trade off with hydraulic safety.	Reduction as a result of acclimation to drier environments. Possibly with stronger changes and being higher in communities with usually wet conditions.	(6)
VLF	Related to water transport capacity of stem. Larger values represent higher possible water conductivity at the partial cost of lower mechanical support. May represent hydraulic efficiency.	Expected reduction to decrease cavitation given lack of water resources under drought.	(7, 8)
VD	Ensure sufficient water supply from the roots to the leaves.<br>Larger vessels diameter is associated with species with rapid water transport to support high photosynthetic rates. Wider vessels may be more susceptible to implosion and have increased risk to embolism and cavitation.	Deciduous species may show wider vessel diameter than evergreen as they avoid dry season cavitation risk.	(6, 9, 10)
ρV	Water transport capacity. Fewer but larger vessels (lower density) may facilitate water transport.	Expected increases in dry environments in association with decrease of vessel size, to maintain water flow and lower cavitation risk	(8)
AreaL	Relevant as a main light capture mechanism. Higher leaf area could result in more leaf transpiration and thus water loss under a drying climate.	Under a drying climate it may increase in deciduous species and expect decreases in evergreens as to limit water loss by transpiration and for increasing cooling.	(11, 12)
SLA	Important for photosynthetic capacity, light capture, water loss, net assimilation rate, leaf life span.	May increase if acquisitive species, e.g. deciduous species, become more abundant with a drying climate.	(13-15)
NL	Essential for metabolic reactions involved in light capture, photosynthetic capacity and growth. Restricted availabilities limit plant carbon acquisition and growth	Drought effects may be compensated if nitrogen fixing species (mainly Fabaceae) become more abundant. May be more dependent on soil conditions than on climate.	(16-18)
PL	Needed nutrient for metabolic reactions that include light capture, related to photosynthetic capacity and growth. Lack of P may limit carbon acquisition and growth	Decreases under a drying climate and possible not strong effect under short term droughts or in wet forests. May be more dependent on soil conditions than on climate.	(16-18)
$Thickness_L$	Trade-off between decreasing water transpiration at the expense of higher construction investment. May decrease under a drying climate as a result of increasing in deciduous species which may tend to have thinner leaves.	It is expected that thicker leaves become more common under larger water deficits for evergreen species but may decreases for acquisitive deciduous species.	(11, 12)

A_{max}	Maximum CO2 assimilation. Index of leaf photosynthetic capacity.	Higher for species with fast resources turnover, e.g. deciduous vs evergreens. Increase with abundance of such species.	(6, 19, 20)
A_{sat}	Saturated photosynthetic rate. Index of leaf photosynthetic capacity.	Declines with higher temperatures and lower precipitation. However, Asat is also dependent on CO2 fertilization and N and P levels.	(6, 19, 20)
Heightmax	Proxy of species position in the vertical light gradient in the forest canopy, with taller species accessing higher light levels than shorter species also given their usually wider crowns.	Taller species that can access more light resources may increase if they can also avoid cavitation risks and have a fast energy turnover as is the case for deciduous species. Otherwise, shorter species with slow growing patterns and with low vessel cavitation risks, e.g. given periods of drought, may become more dominant.	(21, 22)
WD	Relevant for mechanical strengths, stem vulnerability to xylem cavitation.	Expected to be higher in areas with lower water resources, and thus increase with a drying climate.	(15, 22-24)
Phenology –Deciduous/ Evergreens	Deciduous species have low investment in leaf construction, rapid leaf turnover and high photosynthetic capacity. Reduction of water transpiration and avoidance of xylem cavitation are important for their success –drought avoiders.<br><br>Evergreens have high investment in leaf construction, slow leaf turnover, lower photosynthetic capacity –drought resistant	With a drying climate increasing are expected as such species may be better adapted to long and intense periods of drought in comparison to evergreen species, which may tend to decrease in abundance	(25-27)
Guilds	Certain guilds have been shown to be better adapted to droughts, e.g. NPLD than others, e.g. SB	With a drying climate, guilds as NPLD and Pioneers may become more abundant specially if the LA:SA ratio decreases which may negatively affect the abundance of other guilds as Shade Bearers.	(27)
Nitrogen Fixers	Higher productivity given N uptake. Higher leaf nitrogen content and photosynthetic capacity than non-nitrogen fixers. Likely with high rates of photosynthesis over wet periods and accumulation of carbon for foliage production after drought.	Expected increase as such species may have access to limiting resources important for photosynthesis such as nitrogen, which may confer them advantages in a drying climate	(25, 28)

LA:SA: Leaf area-sapwood area ratio; VD: vessel diameter; Kp: hydraulic conductivity; VLF: vessel lumen fraction; pV: vessel density; PL: leaf phosphorous content; SLA: specific leaf area; NL: leaf nitrogen content; AreaL: Leaf area; ThicknessL: leaf thickness; Amax: CO2-saturated assimilation rates; Asat: light-saturated photosynthetic rates; Phenology: deciduous/evergreens; Guilds: NPLD –Non pioneer light demanders, SB–shade bearers, PI–Pioneers, SW–swamp vegetation; Nitrogen fixing capacity: Fixers and not fixers; Heightmax: maximum adult size; WD: wood density.

Supplementary Table 3. Soil and climatic variables included in statistical analysis. For all components we show their summary statistics from across all vegetation plots. The soil components included in the PCA analysis are shown together with their loadings in the selected three PCA axes.

Variable	Component	Mean	Max	Min	SD	PCA Loadings (% variance explained)		
						PC1 (39.4)	PC2 (22.1)	PC3 (14.7)
Soil	Sand (%)	54.12	85.31	28.66	15.85	-0.26	-0.33	0.37
	Silt (%)	25.66	49.98	10.81	10.14	0.28	0.31	0.03
	Clay (%)	20.22	36.09	3.89	10.75	0.12	0.20	-0.58
	N (%)	0.15	0.31	0.06	0.07	0.35	0.15	0.25
	C (%)	1.72	3.82	0.61	0.86	0.32	0.18	0.32
	Total P (mg kg)	137.75	421.90	34.97	88.14	0.22	0.01	-0.41
	Ca (mg kg)	264.21	790.84	32.83	199.23	0.29	-0.41	0.02
	K (mg kg)	53.03	99.20	17.72	22.63	0.32	-0.14	0.08
	Mg (mg kg)	98.54	267.55	20.53	60.93	0.38	-0.05	-0.02
	Na (mg kg)	13.25	144.76	1.50	30.50	0.23	0.18	0.28
	Fe (mg kg)	19.40	77.61	0.42	19.08	-0.08	0.43	0.33
	eCEC (mmol kg)	29.14	55.99	10.99	12.54	0.39	-0.19	-0.01
	pH (KCl)	4.13	5.57	3.47	0.54	0.11	-0.50	0.06
Climate	ΔVPDAbs (kPa)	0.011	0.015	0.006	0.003			
	ΔMCWDAbs (mm)	-17.10	-7.28	-27.53	7.08			
	MCWDFull (mm)	-227.86	-167.36	-300.74	48.28			

Supplementary Table 4. Specification of models fitted in the R statistical environment for each one of the Diversity Metrics (FDis: functional diversity, Simpson: taxonomic diversity and MPD: phylogenetic diversity). The diversity metric was fitted as a response to climatic and soil drivers. For all three diversity metrics the soil PC axes were fitted as quadratic terms. For the MPD models the time between censuses was used as an extra covariate in order to account for its possible role in determining changes in MPD across the different vegetation plots (given the weack but significant correlation between changes in MPD and the years between censuses).

Number	Model summary
1	PC1 + PC2 + PC3 + ΔVPDAbs + MCWDFull + ΔMCWDAbs + PC1*ΔVPDAbs + PC2*ΔVPDAbs + PC3*ΔVPDAbs + PC1*MCWDFull + PC2*MCWDFull + PC3*MCWDFull
2	PC1 + PC2 + PC3 + ΔMCWDAbs + PC1*ΔMCWDAbs + PC2*ΔMCWDAbs + PC3*ΔMCWDAbs + Plot area
3	ΔVPDAbs + MCWDFull + ΔMCWDAbs + Plot area
4	PC1 + PC2 + PC3 + Plot area
5	ΔVPDAbs + Plot area
6	MCWDFull + Plot area
7	ΔMCWDAbs + Plot area
8	PC1 + Plot area
9	PC2 + Plot area
10	PC3 + Plot area
11	PC1 + PC2 + PC3 + ΔVPDAbs + PC1*ΔVPDAbs + PC2*ΔVPDAbs + PC3*ΔVPDAbs + Plot area
12	PC1 + PC2 + PC3 + MCWDFull + PC1*MCWDFull + PC2*MCWDFull + PC3*MCWDFull + Plot area
13	PC1 + PC2 + PC3 + ΔMCWDAbs + PC1*ΔMCWDAbs + PC2*ΔMCWDAbs + PC3*ΔMCWDAbs + Plot area
14	PC1 + ΔMCWDAbs + PC1*ΔMCWDAbs + Plot area
15	PC2 + ΔMCWDAbs + PC2*ΔMCWDAbs + Plot area
16	PC3 + ΔMCWDAbs + PC3*ΔMCWDAbs + Plot area
17	PC1 + ΔVPDAbs + PC1*ΔVPDAbs + Plot area
18	PC2 + ΔVPDAbs + PC2*ΔVPDAbs + Plot area
19	PC3 + ΔVPDAbs + PC3*ΔVPDAbs + Plot area
20	PC1 + MCWDFull + PC1*MCWDFull + Plot area
21	PC2 + MCWDFull + PC2*MCWDFull + Plot area
22	PC3 + MCWDFull + PC3*MCWDFull + Plot area
23	1 + Plot area
24	PC1+PC2 + Plot area
25	PC1+PC3 + Plot area
26	PC2+PC3 + Plot area
27	PC1 + PC2 + MCWDFull + PC1*MCWDFull + PC2*MCWDFull + Plot area
28	PC1 + PC3 + MCWDFull + PC1*MCWDFull + PC3*MCWDFull + Plot area
29	PC2 + PC3 + MCWDFull + PC2*MCWDFull + PC3*MCWDFull + Plot area
30	PC1 + PC2 + ΔMCWDAbs + PC1*ΔMCWDAbs + PC2*ΔMCWDAbs + Plot area
31	PC1 + PC3 + ΔMCWDAbs + PC1*ΔMCWDAbs + PC3*ΔMCWDAbs + Plot area
32	PC2 + PC3 + ΔMCWDAbs + PC2*ΔMCWDAbs + PC3*ΔMCWDAbs + Plot area
33	PC1 + PC2 + ΔVPDAbs + PC1*ΔVPDAbs + PC2*ΔVPDAbs + Plot area
34	PC1 + PC3 + ΔVPDAbs + PC1*ΔVPDAbs + PC3*ΔVPDAbs + Plot area
35	PC2 + PC3 + ΔVPDAbs + PC2*ΔVPDAbs + PC3*ΔVPDAbs + Plot area

Supplementary Table 5. Test of phylogenetic signal on the quantitative traits used. All traits showed significant phylogenetic signal as shown for the significant Blomberg's K values.

Trait	*K*	*P-value*
SLA	0.302	0.031
$Thickness_L$	0.583	0.001
$Area_L$	0.338	0.014
N_L	0.318	0.016
P_L	0.320	0.039
Asat	0.483	0.001
Amax	0.463	0.001
LA:SA	0.608	0.001
VLF	0.720	0.001
VD	0.346	0.009
Kp	0.371	0.023
rV	0.536	0.001
WD	0.557	0.001
$Height_{max}$	0.386	0.002

LA:SA: Leaf area-sapwood area ratio; VD: vessel diameter; Kp: hydraulic conductivity; VLF: vessel lumen fraction; pV: vessel density; PL: leaf phosphorous content; SLA: specific leaf area; NL: leaf nitrogen content; AreaL: Leaf area; ThicknessL: leaf thickness; Amax: CO2-saturated assimilation rates; Asat: light-saturated photosynthetic rates; Heightmax: maximum adult size; WD: wood density.

Supplementary Table 6. Model selection table based on Leave One Out cross-validation information criterion (LOOIC). The best model, the one with the lowest LOOIC and highest ELPD, for each diversity metric is highlighted in grey. The best models are used in subsequent analysis. For more specific information on the terms included in each model see Table S2.

Metric	Model	LOOIC	ELPD LOO	ELPD diff	P LOO	SE LOOIC	SE ELPD LOO	SE P LOO
$\Delta FDis_r$	7	-294.02	147.01	0	3.21	4.07	2.04	0.6
	15	-291.73	145.87	-1.15	4.53	3.12	1.56	0.65
	16	-291.17	145.59	-1.42	4.61	3.96	1.98	0.82
	14	-290.37	145.19	-1.83	5.15	4.67	2.33	1.06
	3	-289.93	144.97	-2.05	5.57	6.65	3.32	1.82
	35	-289.04	144.52	-2.49	5.95	3.77	1.88	0.74
	9	-287.72	143.86	-3.15	2.69	4.65	2.32	0.43
	6	-287.6	143.8	-3.21	2.97	4.62	2.31	0.55
	37	-287.55	143.78	-3.24	6.37	3.87	1.93	1.11
	23	-287.43	143.71	-3.3	2.11	5.41	2.7	0.45
	36	-286.93	143.47	-3.54	6.01	3.8	1.9	0.81
	5	-286.5	143.25	-3.76	3.14	5.75	2.88	0.73
	8	-285.92	142.96	-4.05	2.63	5.27	2.63	0.5
	29	-285.62	142.81	-4.2	3.6	4.87	2.44	0.66
	31	-285.46	142.73	-4.28	3.54	4.69	2.35	0.6
	13	-284.85	142.43	-4.58	8.12	4.21	2.11	1.41
	10	-284.83	142.41	-4.6	3.01	5.31	2.66	0.66
	30	-283.94	141.97	-5.04	3.36	5.33	2.66	0.7
	40	-283.6	141.8	-5.21	6.17	4.66	2.33	1.16
	4	-283.55	141.78	-5.24	4.29	4.67	2.34	0.69
	11	-283.45	141.72	-5.29	7.84	4.15	2.08	1.21
	18	-283.42	141.71	-5.3	4.72	4.89	2.45	0.97
	21	-283.21	141.6	-5.41	4.9	4.23	2.11	0.84
	22	-282.78	141.39	-5.62	4.88	4.5	2.25	0.96
	20	-282.72	141.36	-5.65	4.68	4.43	2.21	0.91
	19	-282.55	141.28	-5.74	4.53	5.38	2.69	0.91
	17	-280.8	140.4	-6.61	5.31	5.98	2.99	1.32
	32	-279.16	139.58	-7.43	6.39	4.29	2.14	1.02
	38	-278.88	139.44	-7.57	6.44	4.55	2.27	1.22
	33	-278.44	139.22	-7.79	5.94	3.78	1.89	0.88
	39	-277.53	138.77	-8.25	6.04	4.85	2.43	1.06
	34	-277.46	138.73	-8.28	7.3	4.66	2.33	1.45
	12	-275.62	137.81	-9.2	9.54	5.16	2.58	1.67
	1	-155.19	77.59	-69.42	9.61	9.47	4.74	3.34
	2	-139.64	69.82	-77.19	21.15	5.87	2.94	2.72
$\Delta Simpson_r$	7	-274.38	137.19	0	3.36	5.58	2.79	0.77
	30	-272.44	136.22	-0.97	6.74	5.29	2.64	1.27
	15	-271.51	135.76	-1.43	5.08	4.76	2.38	0.92
	16	-271.46	135.73	-1.46	5.23	6.39	3.2	1.23
	5	-271.46	135.73	-1.46	3.46	5.19	2.59	0.79

	6	-271.27	135.64	-1.55	3.2	5.66	2.83	0.76
	23	-270.75	135.38	-1.81	2.75	5.8	2.9	0.76
	17	-270.17	135.09	-2.1	4.78	4.6	2.3	0.89
	19	-270.13	135.07	-2.12	4.92	4.69	2.34	0.96
	3	-269.69	134.85	-2.34	5.78	5.69	2.85	1.4
	9	-269.2	134.6	-2.59	3.49	5.36	2.68	0.91
	14	-269.08	134.54	-2.65	5.95	6.37	3.18	1.45
	8	-269.06	134.53	-2.66	3.81	5.59	2.8	0.94
	21	-268.98	134.49	-2.7	4.51	4.34	2.17	0.73
	32	-268.83	134.42	-2.77	6.85	4.74	2.37	1.14
	10	-268.55	134.27	-2.92	3.75	5.35	2.67	0.95
	20	-268.22	134.11	-3.08	4.95	5.05	2.52	1.04
	34	-268.07	134.03	-3.16	6.57	4.75	2.37	1.14
	18	-267.85	133.93	-3.26	4.82	4.93	2.47	1.1
	27	-267.75	133.88	-3.31	5.75	4.51	2.25	0.98
	24	-267.6	133.8	-3.39	4.31	5.01	2.5	0.99
	31	-267.34	133.67	-3.52	7.17	6.2	3.1	1.43
	13	-267.19	133.6	-3.59	8.84	4.66	2.33	1.31
	25	-267.18	133.59	-3.6	4.65	5	2.5	1.09
	26	-267.03	133.51	-3.68	4.54	5.24	2.62	1.1
	22	-266.83	133.41	-3.78	5.18	5.34	2.67	1.32
	4	-265.55	132.77	-4.42	5.25	4.71	2.36	1.11
	33	-265.54	132.77	-4.42	6.34	4.45	2.22	0.98
	35	-265.27	132.63	-4.56	6.57	4.37	2.18	1.11
	29	-263.31	131.66	-5.53	6.37	4.22	2.11	1.15
	11	-262.99	131.49	-5.7	8.18	4.43	2.22	1.15
	1	-262.58	131.29	-5.9	8.36	5.53	2.77	1.65
	12	-262.38	131.19	-6	7.88	4.93	2.46	1.52
	28	-261.54	130.77	-6.42	7.42	5.88	2.94	1.85
	2	-257.21	128.6	-8.59	19.7	5.02	2.51	2.26
	2	-36.94	18.47	0	21.03	5.19	2.59	2.26
	30	-19.64	9.82	-8.65	4.09	5.04	2.52	0.92
	8	-19.42	9.71	-8.76	3.2	5.42	2.71	0.84
	14	-17.74	8.87	-9.6	4.61	4.52	2.26	0.76
	11	-17.65	8.83	-9.64	9.21	4.61	2.31	1.61
	17	-16.89	8.44	-10.02	4.48	5.27	2.64	0.89
	4	-16.57	8.28	-10.18	5.41	5.17	2.58	1.14
ΔMPD_r	40	-16.56	8.28	-10.19	7.12	4.97	2.48	1.32
	29	-16.07	8.03	-10.44	4.7	5.5	2.75	1.23
	38	-15.28	7.64	-10.83	6.8	6.42	3.21	1.53
	20	-14.92	7.46	-11.01	4.6	4.82	2.41	0.93
	33	-14.89	7.45	-11.02	6.9	5.54	2.77	1.4
	35	-13.6	6.8	-11.67	6.94	7.11	3.56	2.09
	1	-13.55	6.77	-11.69	8.36	6.05	3.03	1.79
	36	-12.29	6.14	-12.32	7.63	5.62	2.81	1.83
	18	-11.78	5.89	-12.58	5.78	7.01	3.5	1.56

12	-11.1	5.55	-12.92	9.15	5.53	2.76	1.71
39	-10.77	5.39	-13.08	8.07	6.49	3.25	2.09
5	-10.65	5.32	-13.14	3.16	5.15	2.58	0.7
32	-10.45	5.23	-13.24	6.92	5.53	2.76	1.6
23	-10.16	5.08	-13.39	2.11	4.23	2.12	0.46
10	-9.42	4.71	-13.76	2.96	4.03	2.02	0.54
16	-9.03	4.51	-13.95	5.4	7.17	3.58	1.78
6	-8.47	4.23	-14.23	3.56	5.27	2.64	0.94
13	-7.88	3.94	-14.53	10.32	6.48	3.24	2.38
7	-7.73	3.87	-14.6	3.24	4.28	2.14	0.75
37	-6.96	3.48	-14.99	8.06	6.37	3.18	2.02
9	-6.77	3.38	-15.08	3.78	5.09	2.55	1.08
3	-6.73	3.37	-15.1	4.46	4.9	2.45	0.81
31	-6	3	-15.47	4.54	4.84	2.42	1.04
19	-5.15	2.57	-15.89	6.05	6.44	3.22	1.68
15	-4.89	2.44	-16.02	6.07	6.24	3.12	1.81
21	-4.23	2.11	-16.35	6.26	6.75	3.37	1.84
22	-2.09	1.05	-17.42	6.88	6.91	3.46	2.15
34	1.98	-0.99	-19.46	10.2	9	4.5	3.24

LOOIC: Leave one out information criterion; ELPD LOO: Leave one out expected log predicted density; ELPD diff: difference in expected log predicted density; P LOO: Effective number of parameters; SE LOOIC: standard error of LOOIC; SE ELPD LOO: Standard Error of ELPD; SE P LOO: Standard error of P.

Supplementary Table S7. Linear regression results for the second best models, based on the leave one out cross-validation information criterion (LOOIC), explaining the functional (FDis), taxonomic (Simpson) and phylogenetic (MPD) diversity changes as a function of climatic and soil drivers. Several different models were fitted (see Supplementary Table 4 and Supplementary Table S6) to investigate the drivers of changes of each diversity facet. The most parsimonious model is shown in Table 1.

Metric	Parameter	Median	HDI low	HDI high	ROPE %	Rhat	LOOIC
$\Delta FDis_r$	*Intercept*	3.97E-05	-4.30E-05	1.20E-04	0.30	1.00	-291.73
	PC2	2.16E-05	-2.89E-05	7.12E-05	0.51	1.00	
	ΔMCWDAbs	1.36E-04	5.43E-05	2.18E-04	0.00	1.00	
	Plot area	-8.42E-05	-1.62E-04	-5.48E-06	0.07	1.00	
	PC2:ΔMCWDAbs	3.62E-05	-1.56E-05	8.86E-05	0.33	1.00	
$\Delta Simpson_r$	*Intercept*	2.76E-04	1.21E-04	4.21E-04	0.00	1.00	-272.44
	PC1	7.48E-05	6.24E-06	1.51E-04	0.12	1.00	
	PC2	-1.03E-04	-2.01E-04	-1.63E-05	0.05	1.00	
	ΔMCWDAbs	2.67E-04	1.01E-04	4.21E-04	0.00	1.00	
	Plot area	1.40E-04	-1.49E-05	2.89E-04	0.08	1.00	
	PC1:ΔMCWDAbs	8.20E-05	1.64E-05	1.50E-04	0.08	1.00	
	PC2:ΔMCWDAbs	-1.14E-04	-2.02E-04	-3.32E-05	0.00	1.00	
ΔMPD_r	*Intercept*	-0.15	-0.20	-0.10	0.00	1.00	-19.64
	PC1	-0.05	-0.07	-0.03	0.00	1.00	
	PC3	0.03	0.00	0.07	0.21	1.00	
	Plot area	0.05	-0.01	0.10	0.15	1.00	

HDI: Highest density interval; ROPE: region of practical equivalence to test the importance of parameters; Rhat: potential scale reduction statistic.

References

1. Hallett LM, Hsu JS, Cleland EE, Collins SL, Dickson TL, Farrer EC, et al. Biotic mechanisms of community stability shift along a precipitation gradient. Ecology. 2014;95(6):1693-700.

2. Gross K, Cardinale BJ, Fox JW, Gonzalez A, Loreau M, Wayne Polley H, et al. Species richness and the temporal stability of biomass production: A new analysis of recent biodiversity experiments. Am Nat. 2013;183(1):1-12.

3. R Core Team. R: A language and environment for statistical computing. R foundation for statistical computing, vienna, austria. available online at https://Www.R-project.org/. 2019;3.4.1.

4. Gotsch SG, Geiger EL, Franco AC, Goldstein G, Meinzer FC, Hoffmann WA. Allocation to leaf area and sapwood area affects water relations of co-occurring savanna and forest trees. Oecologia. 2010;163(2):291-301.

5. Rosas T, Mencuccini M, Barba J, Cochard H, Saura-Mas S, Martínez-Vilalta J. Adjustments and coordination of hydraulic, leaf and stem traits along a water availability gradient. New Phytol. 2019.

6. Pineda-García F, Paz H, Meinzer FC, Angeles G. Exploiting water versus tolerating drought: Water-use strategies of trees in a secondary successional tropical dry forest. Tree Physiol. 2015;36(2):208-17.

7. Zanne AE, Westoby M, Falster DS, Ackerly DD, Loarie SR, Arnold SE, et al. Angiosperm wood structure: Global patterns in vessel anatomy and their relation to wood density and potential conductivity. Am J Bot. 2010;97(2):207-15.

8. Hietz P, Rosner S, Hietz-Seifert U, Wright SJ. Wood traits related to size and life history of trees in a panamanian rainforest. New Phytol. 2017;213(1):170-80.

9. Hacke UG, Sperry JS. Functional and ecological xylem anatomy. Perspect Plant Ecol Evol Syst. 2001;4(2):97-115.

10. Schreiber SG, Hacke UG, Hamann A. Variation of xylem vessel diameters across a climate gradient: Insight from a reciprocal transplant experiment with a widespread boreal tree. Funct Ecol. 2015;29(11):1392-401.

11. Lázaro-Nogal A, Matesanz S, Godoy A, Pérez-Trautman F, Gianoli E, Valladares F. Environmental heterogeneity leads to higher plasticity in dry-edge populations of a semi-arid chilean shrub: Insights into climate change responses. J Ecol. 2015;103(2):338-50.

12. Greenwood S, Ruiz-Benito P, Martínez-Vilalta J, Lloret F, Kitzberger T, Allen CD, et al. Tree mortality across biomes is promoted by drought intensity, lower wood density and higher specific leaf area. Ecol Lett. 2017;20(4):539-53.

13. Cornelissen JH, PÉREZ-HARGUINDEGUY N, Díaz S, Grime JP, Marzano B, Cabido M, et al. Leaf structure and defence control litter decomposition rate across species and life forms in regional floras on two continents. New Phytol. 1999;143(1):191-200.

14. Poorter L, McDonald I, Alarcón A, Fichtler E, Licona J, Peña-Claros M, et al. The importance of wood traits and hydraulic conductance for the performance and life history strategies of 42 rainforest tree species. New Phytol. 2010;185(2):481-92.

15. Lohbeck M, Poorter L, Paz H, Pla L, van Breugel M, Martínez-Ramos M, et al. Functional diversity changes during tropical forest succession. Perspect Plant Ecol Evol Syst. 2012;14(2):89-96.

16. Elser JJ, Bracken ME, Cleland EE, Gruner DS, Harpole WS, Hillebrand H, et al. Global analysis of nitrogen and phosphorus limitation of primary producers in freshwater, marine and terrestrial ecosystems. Ecol Lett. 2007;10(12):1135-42.

17. Reich PB, Oleksyn J, Wright IJ, Niklas KJ, Hedin L, Elser JJ. Evidence of a general 2/3-power law of scaling leaf nitrogen to phosphorus among major plant groups and biomes. Proceedings of the Royal Society of London B: Biological Sciences. 2010;277(1683):877-83.

18. He M, Dijkstra FA. Drought effect on plant nitrogen and phosphorus: A meta-analysis. New Phytol. 2014;204(4):924-31.

19. Sobrado M. Cost-benefit relationships in deciduous and evergreen leaves of tropical dry forest species. Funct Ecol. 1991:608-16.

20. Mielke MS, Almeida AFd, Gomes FP. Photosynthetic traits of five neotropical rainforest tree species: Interactions between light response curves and leaf-to-air vapour pressure deficit. Brazilian Archives of Biology and Technology. 2005;48(5):815-24.

21. Poorter L, Bongers F, Sterck FJ, Wöll H. Beyond the regeneration phase: Differentiation of height–light trajectories among tropical tree species. J Ecol. 2005;93(2):256-67.

22. Poorter L, Hawthorne W, Bongers F, Sheil D. Maximum size distributions in tropical forest communities: Relationships with rainfall and disturbance. J Ecol. 2008;96(3):495-504.

23. Chave J, Coomes D, Jansen S, Lewis SL, Swenson NG, Zanne AE. Towards a worldwide wood economics spectrum. Ecol Lett. 2009;12(4):351-66.

24. Markesteijn L, Poorter L, Paz H, Sack L, Bongers F. Ecological differentiation in xylem cavitation resistance is associated with stem and leaf structural traits. Plant, Cell Environ. 2011;34(1):137-48.

25. Poorter L, Markesteijn L. Seedling traits determine drought tolerance of tropical tree species. Biotropica. 2008;40(3):321-31.

26. Fauset S, Baker TR, Lewis SL, Feldpausch TR, Affum-Baffoe K, Foli EG, et al. Drought-induced shifts in the floristic and functional composition of tropical forests in ghana. Ecol Lett. 2012;15(10):1120-9.

27. Gvozdevaite A, Oliveras I, Domingues TF, Peprah T, Boakye M, Afriyie L, et al. Leaf-level photosynthetic capacity dynamics in relation to soil and foliar nutrients along forest–savanna boundaries in ghana and brazil. Tree Physiol. 2018;38(12):1912-25.

28. Eamus D, Prior L. Ecophysiology of trees of seasonally dry tropics: Comparisons among phenologies. . 2001.
